# Development of an efficient *in vitro* plant regeneration system using true leaf explants of processing tomato (*Solanum lycopersicum* L.) ‘Ligeer 87-5’

**DOI:** 10.7717/peerj.21520

**Published:** 2026-07-14

**Authors:** JiangYuan Zhang, HongRun Zhou, HaoRan Kan, XinXin Jin, Jing Ye, Jie Zhao

**Affiliations:** 1 Key Laboratory of Xinjiang for Oasis Agricultural Pests Management and Plant Protection Resources Utilization, Agricultural College, Shihezi University, Shihezi, Xinjiang, China; 2 Laboratory of Forestry Department, College of Urban and Environmental Sciences, Shihezi University, Shihezi, Xinjiang, China

**Keywords:** Tissue culture, Tomato, Leaf explants, Embryogenesis

## Abstract

Tomato (*Solanum lycopersicum* L.) is one of the most economically important horticultural crops in China, and Xinjiang is the main region in China for processing tomato production. The establishment of a robust in vitro regeneration system is a fundamental prerequisite for genetic improvement and cultivar innovation in this crop. Among the factors influencing regeneration efficiency, exogenously applied plant growth regulators (PGRs) are the most important because they regulate endogenous phytohormone homeostasis and signaling pathways. In this study, true leaf explants of the processing tomato Ligeer 87-5 were used to optimize combinations of 6-benzyladenine (6-BA) and indole-3-acetic acid (IAA) and to establish an efficient in vitro regeneration system. Full strength MS medium supplemented with 2 mg/L 6-BA and 0.2 mg/L IAA was optimal for callus induction and organogenic competence, resulting in a callus induction rate of 97.00%, an embryogenic cell incidence of 63.88%, and an adventitious bud formation rate of 21.33% after 20 days; the budding rate further increased to 63.00% after 30 days. For rapid shoot induction, a medium composed of MS + 1 mg/L 6-BA + 0.2 mg/L IAA yielded the highest adventitious bud formation rate, reaching 89.00% after 10 days. Rooting was most effective on MS medium supplemented with 0.2 mg/L IAA, achieving a 100% rooting rate after 21 days, an average root length of 11.76 cm, a 76.67% acclimatization rate after 28 days, and a 100% survival rate after hardening and transplantation. Collectively, the stage-specific application of different media substantially improved true-leaf regeneration efficiency in processing tomato plants, providing a robust platform for future genetic transformation and molecular breeding.

## Introduction

Processing tomato is a globally important horticultural and industrial crop serving as the primary raw material for value-added products such as tomato paste, diced tomato, and tomato powder, with an annual global planting area exceeding five million hectares (Food Agriculture Organization of the United Nations, 2024). China is the world’s leading producer and exporter of processing tomato products, and the Xinjiang region alone contributes more than 80% of the national processing tomato acreage and about one fifth of global annual output. Consequently, processing tomato has become a leading pillar of the regional agricultural economy (Department of Finance of the Xinjiang Uygur Autonomous Region, 2025).

Despite its economic importance, processing tomato production is increasingly constrained by multiple biotic and abiotic challenges. Frequent outbreaks of soil-borne diseases, including late blight and bacterial wilt, together with persistent losses caused by viral diseases and insect pests, and increasing drought and soil salinization in major growing regions, have substantially constrained yield stability and quality improvement (Zhang, 2025; Zheng et al., 2025). At the same time, industrial and market standards for processing quality traits such as soluble solids content, lycopene concentration, and product viscosity, have become increasingly stringent (Cui et al., 2023). Traditional cross-breeding is limited by long cycles, low efficiency, and difficulty breaking linkage drag for unfavorable traits, so it can no longer meet the urgent need to rapidly improve stress resistance and quality in processing tomato varieties (Cui et al., 2023).

Plant tissue culture is an efficient method for improving crop genetics, conserving germplasm, and enabling commercial micropropagation. A simple, effective regeneration system is therefore a fundamental prerequisite for these applications (Kulus, 2019; Hamdan et al., 2021; Ben-Amar, Allel & Bouamama-Gzara, 2024). Among the factors governing *in vitro* morphogenesis, exogenous plant growth regulators (PGRs) are widely used to manipulate *in vitro* cultures. They can alter the balance and signaling of endogenous hormones, which are the central regulators of cell division, dedifferentiation, and redifferentiation in plants (Long et al., 2022). Consequently, the concentration and combination of exogenous PGRs substantially affect regeneration efficiency (Bhatia et al., 2004).

Tomato is not only an economically important crop but also a valuable dietary source of nutrients such as lycopene, β-carotene, and flavonoids. The establishment of an efficient regeneration system is therefore critical for supporting genetic transformation, gene editing, and target trait improvements in this species (Gerszberg et al., 2015; Sharma, Thakur & Negi, 2019). Previous studies have demonstrated that a wide range of tomato explants, including apical meristems, cotyledons, petioles, stem segments, true leaves, anthers, and inflorescences, are capable of producing adventitious shoots *in vitro* (Rosa et al., 2022). Notably, explant type is a major determinant of regeneration success in tomato plants.

Regeneration systems based on tomato cotyledons and hypocotyls are widely employed as explants, but they require large numbers of seedlings to provide sufficient explant material. Moreover, explants collected at the seed-germination stage are strongly influenced by seedling age and physiological status, which can introduce considerable variation in *in vitro* culture responses (Cortina & Culiañez Macià, 2004; Murugan et al., 2025; Ahmed et al., 2023). In contrast, true leaf tissues offer several practical advantages for *in vitro* culture, including ease of excision and an abundant supply of explants. A few donor plants can produce large amounts of callus, thereby improving experimental consistency and reducing reliance on repeated seed germination. Consequently, regeneration protocols that use true leaves as explants can preserve parental traits and avoid the trait segregation associated with seed-based propagation, making them a useful platform for propagating elite lines and studying gene function. However, true leaf tissues are more terminally differentiated and have a reduced innate capacity for *in vitro* dedifferentiation and regeneration. As a result, leaf-based culture systems suffer from low induction efficiency and strong genotype dependence (Gerszberg et al., 2015).

An efficient de novo *in vitro* regeneration system is a prerequisite for genetic improvement and functional genomics in processing tomato. Without standardized protocols for *in vitro* propagation across different tomato types, developing a true-leaf tissue culture system for processing tomatoes is essential to improve breeding efficiency and expand biotechnological applications. The processing tomato cultivar ‘Ligeer 87-5’ is an early-maturing, determinate variety characterized by strong branching and deep-red fruit. As a homozygous, genetically stable conventional cultivar, it is valued for excellent processing quality, high yield, and robust disease resistance (Chen et al., 2024). In the present study, a highly efficient and stable true-leaf regeneration system was established using true leaf explants of Ligeer 87-5. Specifically, concentrations of the exogenous PGRs, 6-benzyladenine (6-BA), and indole-3-acetic acid (IAA) were optimized at successive tissue culture stages to induce callus formation, adventitious shoot regeneration, and adventitious root formation from leaf explants.

## Materials & Methods

### Seed sterilization and culture

Healthy seeds of tomato cultivar Ligeer 87-5 were soaked in sterilized water for 16 h, surface-sterilized in 75% ethanol for 1 min, treated with 10% NaClO for 30 min, and finally rinsed 3–4 times with sterile water. The sterilized seeds were inoculated onto 41.4 g/L MS modified medium (MMM, manufactured by Coolaber, PM10121-307), containing 4.3 g/L MS basal salts, 0.1031 g/L vitamins, 30 g/L sucrose, and 7 g/L agar. The pH of the medium was adjusted to 5.8 prior to autoclaving. Cultures were initially maintained in the dark at 25 °C with 80% relative humidity for three days, then were transferred to a 16 h light/8 h dark photoperiod for 20 days under a photosynthetic photon flux density (PPFD) of approximately 64 µmol m^−^^2^ s^−^^1^. At the end of this culture period, seedlings had developed 8–9 true leaves.

### Callus induction and adventitious bud differentiation

True leaves were excised and cut into 1 cm   ×  1 cm explants, which were cultured on MS medium supplemented with 6-BA (2 or 3 mg/L) and IAA (0.1, 0.2, or 0.3 mg/L). Each treatment consisted of 30 explants and three independent biological replicates. Cultures were maintained at 25 °C, 80% relative humidity, under a 16 h light/8 h dark photoperiod for 30 days. Explant morphology was assessed every five days, and medium was refreshed with the same formulation every 10 days. Callus induction rate = (Number of explants with callus/Total number of explants) × 100%, and was recorded on days 10, 15, and 20 post-cultivation. Budding rate = (Number of callus pieces with buds/Total number of callus pieces) × 100%, and was recorded on days 20, 25, and 30 post-cultivation.

### Microscopic observation of callus tissue

Paraffin sections were prepared from callus tissues collected after 20 days of cultivation, with three calli sampled per medium. Samples were fixed in formalin: acetic acid: 70% ethanol (FAA) at a ratio of 1:1:18, dehydrated through a graded ethanol–xylene series, embedded in paraffin, and sectioned at 5 µm using a sliding microtome. The sections were stained with fast green and examined with an optical microscope (OLYMPUS BX61). Embryogenic cells were identified according to established cytological criteria, including prominent nuclei, dense cytoplasm, high nucleus-to-cytoplasm ratio, tight intercellular arrangement, and absence of large vacuoles (Gaj, 2004). Cytological features were observed and recorded. Five random, independent visual fields were selected from each section for counts of embryogenic cells and total cells.

Embryogenic rate = (Number of embryogenic cells/Total number of counted cells) ×100%.

### Adventitious bud elongation

Callus pieces bearing adventitious buds were transferred to MS medium supplemented with 6-BA (1 or 1.5 mg/L) and IAA (0.1, 0.2, or 0.3 mg/L). Each treatment consisted of 30 callus pieces and was performed in three independent biological replicates. Regenerated shoots were cultured at 25 °C under a 16 h light/8 h dark photoperiod. For acclimation, plantlets were maintained in a growth chamber at 25 °C and an initial relative humidity of 90–95% for 10 days. Morphological changes in the adventitious buds were recorded at 5-day intervals.

Germination rate = (Number of callus pieces with buds/Total number of callus pieces) ×100%, and was recorded on days 5 and 10.

### *In vitro* rooting

Elongated adventitious shoots were transferred individually to MS medium supplemented with IAA at 0.1, 0.2, or 0.3 mg/L for root induction, with one shoot cultured per vessel. Each treatment comprised three replicates, with 15 vessels per replicate. Cultures were maintained at 25 °C and 80% relative humidity under a 16 h light/8 h dark photoperiod for 28 days. Morphological changes in buds and roots were assessed at 7-day intervals, and root length and rooting rate were recorded throughout the culture period. Rooting rate = (Number of shoots with roots/Total number of shoots) ×100%. For tomato seedlings on the different rooting media, the hardening rate was defined as (Number of normal plantlets/Total number of shoots) ×100%.

### Acclimatization of *in vitro* plantlets

Regenerated plantlets were acclimated to ex vitro conditions using a stepwise hardening-off protocol. During the first seven days, plantlets were maintained in a growth chamber at 25 °C, 80% relative humidity, and 50% shading under a 16 h light/8 h dark photoperiod. Over the subsequent 14 days, environmental parameters were adjusted gradually: light intensity was increased to 64 µmol m^−^^2^ s^−^^1^, humidity was reduced to 60%, and temperature remained at 25 °C. Ventilation time was progressively extended from 2 h/day until plants were fully exposed. Survival rate was determined at the end of the domestication stage and defined as (Number of survived plantlets/Total number of plantlets) × 100%.

### Statistical analysis

Data were summarized and calculated in Microsoft Excel 2019 (Version 16.0) and analyzed in IBM SPSS Statistics (Version 27). Callus induction rate, embryogenic rate, budding rate, germination rate, rooting rate, root length, and hardening rate were reported as means ± standard deviation (SD). Homogeneity of variance was assessed with Levene’s test, after which group comparisons were performed using Duncan’s or Tamhane’s T2 test at *p* = 0.05. During the callus, bud induction, and bud elongation stages, the effects of IAA, 6-BA, and their interaction were evaluated with the F-statistic and *η*^2^. The F-statistic is the ratio of the mean square for the treatment effect to the mean square of the error and tests the overall significance of a factor. A larger F-value indicates that between-group variation exceeds within-group random variation. The *η*^2^ denotes the proportion of total variance in the dependent variable explained by a given independent variable, and values closer to 1 indicate a stronger effect. The *p*-value associated with the F-test indicates whether the observed effect is statistically significant.

## Results

### Effects of exogenous 6-BA and IAA concentration combinations on callus induction phase

The effects of various culture media on callus induction are summarized in Table 1. In all treatments, the callus induction rate increased progressively with culture duration. The highest callus induction rate, which reached an average of 97.00% at the 20th day, was obtained on Medium 2, which contained 2 mg/L 6-BA and 0.2 mg/L IAA. During the callus induction phase, the concentrations of both 6-BA and IAA had a significant impact on the callus induction rate (*F*_6-BA_ = 64.06, *η*^2^ = 78.1%, *p* < 0.001; *F*_IAA_ = 16.06, *η*^2^ = 64.1%, *p* < 0.001), and 6-BA was the primary influencing factor.

**Figure 1:**
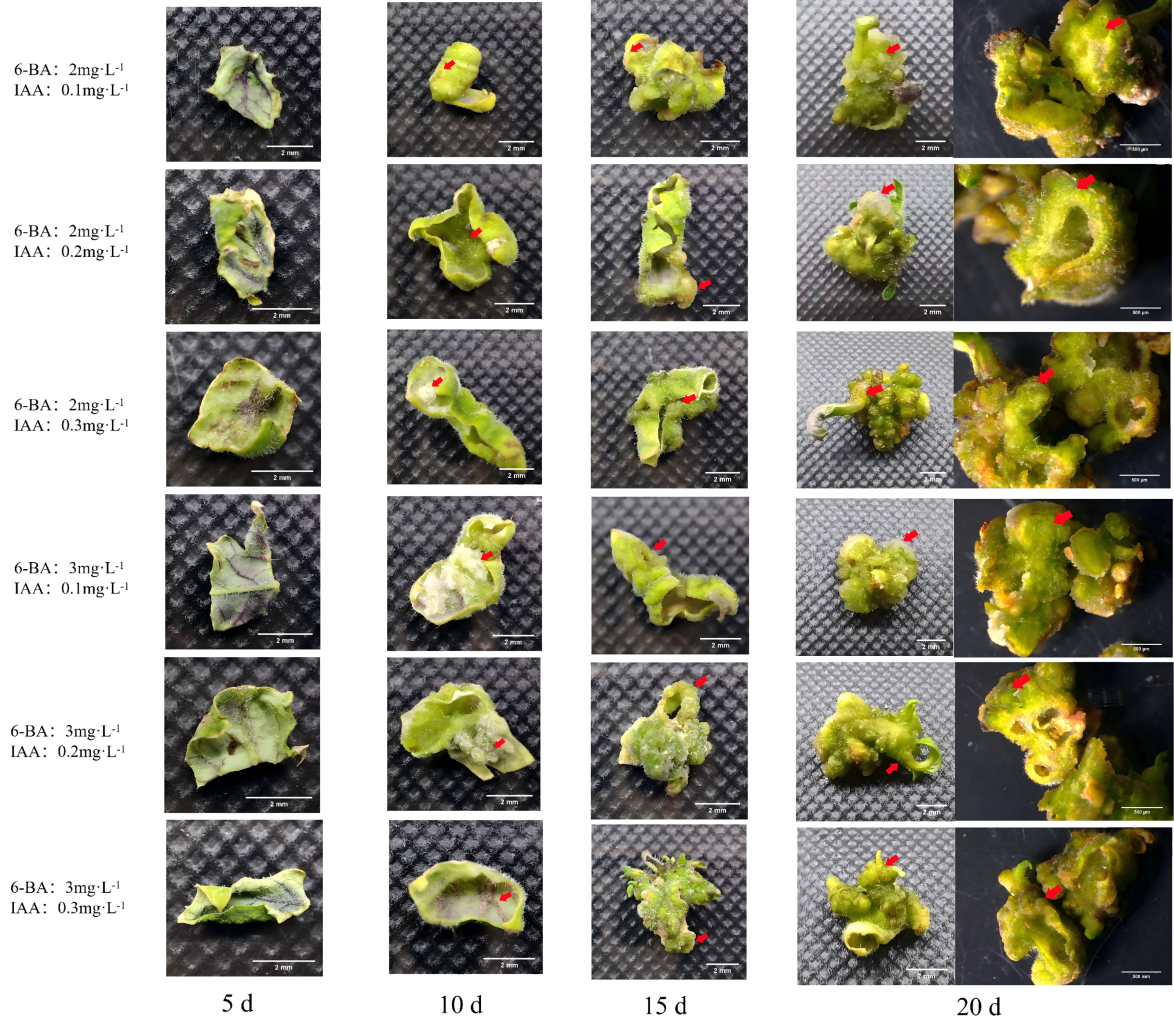
Effects of exogenous 6-BA and IAA concentration combinations on callus induction. The explants were inoculated onto MS modified media containing varying concentrations of 6-benzyladenine (6-BA; 2 and 3 mg/L) and indole-3-acetic acid (IAA, 0.1, 0.2, and 0.3 mg/L) for 20 days, and images were captured every five days. The callus is indicated by the red arrow.

**Figure 2:**
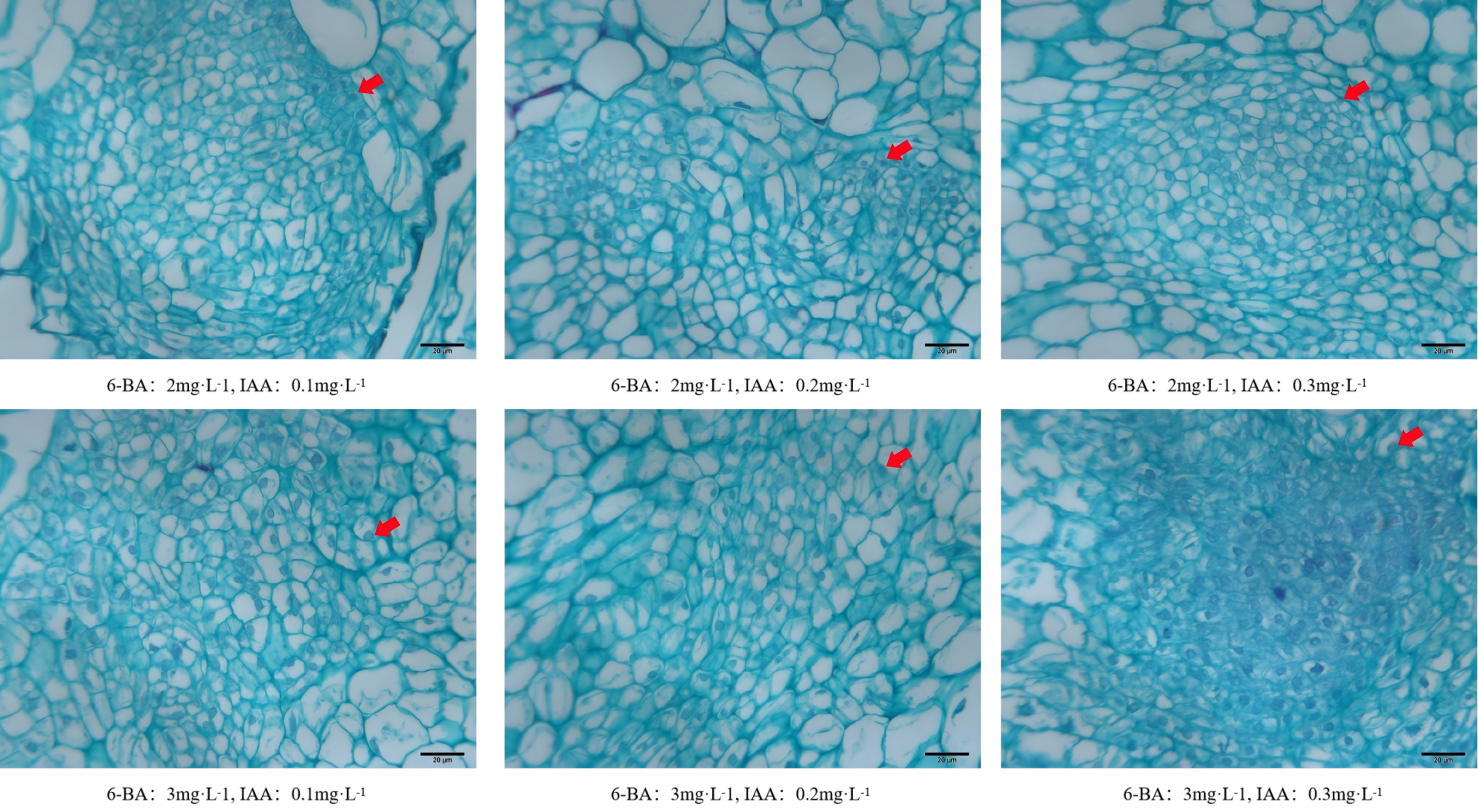
Paraffin sections of callus tissue on day 20. The explants inoculated onto MS modified media containing varying concentrations of 6-BA (2 and 3 mg/L) and IAA (0.1, 0.2, and 0.3 mg/L) for 20 days. The callus tissue was cut to prepare paraffin sections. The nucleus of the embryogenic cell is indicated by the red arrow.

As shown in Fig. 1, the first visible callus appeared at day 10, proliferated significantly by day 15, and developed into dense callus masses by day 20. Histological analysis of 20-day-old callus using paraffin sections (Fig. 2) revealed the coexistence of embryogenic and non-embryogenic cell populations. The incidence of embryogenic cells varied significantly across the different callus induction media. Notably, the interaction between 6-BA and IAA had a predominant effect on the incidence of embryogenic cells, which were identified based on the classic morphological criteria for plant somatic embryogenesis (*F*_6-BA×IAA_ = 33.80, *η*^2^ = 84.9%, *p* < 0.001). Medium 2 and Medium 4 exhibited significantly higher embryogenic rates (63.88% and 67.66%, respectively) compared to other media, with no significant difference between them. After assessing the callus induction rate and embryogenic rate during the callus induction phase, Medium 2, which comprises MS media, 2 mg/L 6-BA, and 0.2 mg/L IAA, was identified as the optimal culture medium.

**Table 1:** Callus induction rate, cellular embryogenic rate, and budding rate.

Medium number	Hormone concentration (mg/L)		Callus induction rate (%)			Embryogenic rate (%)	Budding rate (%)		
	6-BA	IAA	Day 10	Day 15	Day 20	Day 20	Day 20	Day 25	Day 30
1	2	0.1	17.00 ± 2.00 c	70.00 ± 2.31 c	86.00 ± 2.31 b	53.19 ± 1.34 b	8.00 ± 0.00 c	24.00 ± 4.00 c	45.00 ± 3.83 c
2	2	0.2	13.00 ± 2.00 d	89.00 ± 2.00 a	97.00 ± 3.83 a	63.88 ± 3.05 a	21.33 ± 2.31 a	33.33 ± 2.31 b	63.00 ± 2.00 a
3	2	0.3	13.00 ± 2.00 d	74.00 ± 2.31 b	84.00 ± 3.27 bc	55.64 ± 3.64 b	17.33 ± 2.31 b	34.67 ± 2.31 b	44.00 ± 3.27 c
4	3	0.1	21.00 ± 2.00 b	58.00 ± 2.31 d	80.00 ± 4.62 c	67.66 ± 4.32 a	4.00 ± 0.00 d	17.33 ± 2.31 d	52.00 ± 3.27 b
5	3	0.2	18.00 ± 2.31 c	49.00 ± 3.83 f	80.00 ± 3.27 c	52.79 ± 1.12 b	9.33 ± 2.31 c	32.00 ± 0.00 b	52.00 ± 3.27 b
6	3	0.3	26.00 ± 2.31 a	54.00 ± 2.31 e	74.00 ± 2.31 d	54.19 ± 0.15 b	21.33 ± 2.31 a	41.33 ± 2.31 a	55.00 ± 2.00 b

**Notes.**

Data are presented as mean ± SD.

Different lowercase letters within a column indicate significant differences (*p* < 0.05).

### Effects of exogenous 6-BA and IAA concentration combinations on shoot growth phase

The same 6-BA and IAA combinations used for callus induction were initially applied during adventitious bud induction. The resulting budding rates during the bud induction stage across various culture media are presented in Table 1. By day 20 of callus induction, small adventitious bud primordia appeared in all treatment groups. Thereafter, the budding rate of adventitious buds, indicating the percentage of callus pieces developing adventitious buds, increased progressively in every treatment. Among the tested media, Medium 2, comprising the MS media with 2 mg/L 6-BA and 0.2 mg/L IAA, achieved the highest average budding rate of 63.00% by day 30 of culture.

Subsequently, the callus underwent transfer to different media for bud elongation experiments, where the 6-BA concentration decreased while the IAA concentration remained constant, as detailed in Table 2. After an additional 10 days of culture, Medium 8 exhibited the highest average germination rate of 89%, significantly differing from the other media. Throughout the continuous shoot development process, the effects of exogenous PGR treatments on shoot phenotypic outcomes varied dynamically. The interaction between exogenous 6-BA and IAA significantly influenced adventitious bud differentiation (*F*_6-BA×IAA_ = 30.15, *η*^2^ = 77.0%, *p* < 0.001), while the concentration of exogenous 6-BA primarily affected subsequent shoot elongation (*F*_6-BA_ = 76.8, *η*^2^ = 81.0%, *p* < 0.001).

**Table 2:** Germination rates in bud elongation induction stage.

Medium number	Hormone Concentration (mg/L)		Germination rate (%)	
	6-BA	IAA	Day 5	Day 10
7	1	0.1	69.00 ± 2.00 b	81.00 ± 2.00 b
8	1	0.2	80.00 ± 4.62 a	89.00 ± 5.03 a
9	1	0.3	62.00 ± 2.31 c	77.00 ± 2.00 bc
10	1.5	0.1	63.00 ± 2.00 c	73.00 ± 2.00 c
11	1.5	0.2	70.00 ± 2.31 b	74.00 ± 2.31 c
12	1.5	0.3	64.00 ± 0.00 c	68.00 ± 3.27 d

**Notes.**

Data are presented as mean ± SD.

Different lowercase letters within a column indicate significant differences (*p* < 0.05).

**Figure 3:**
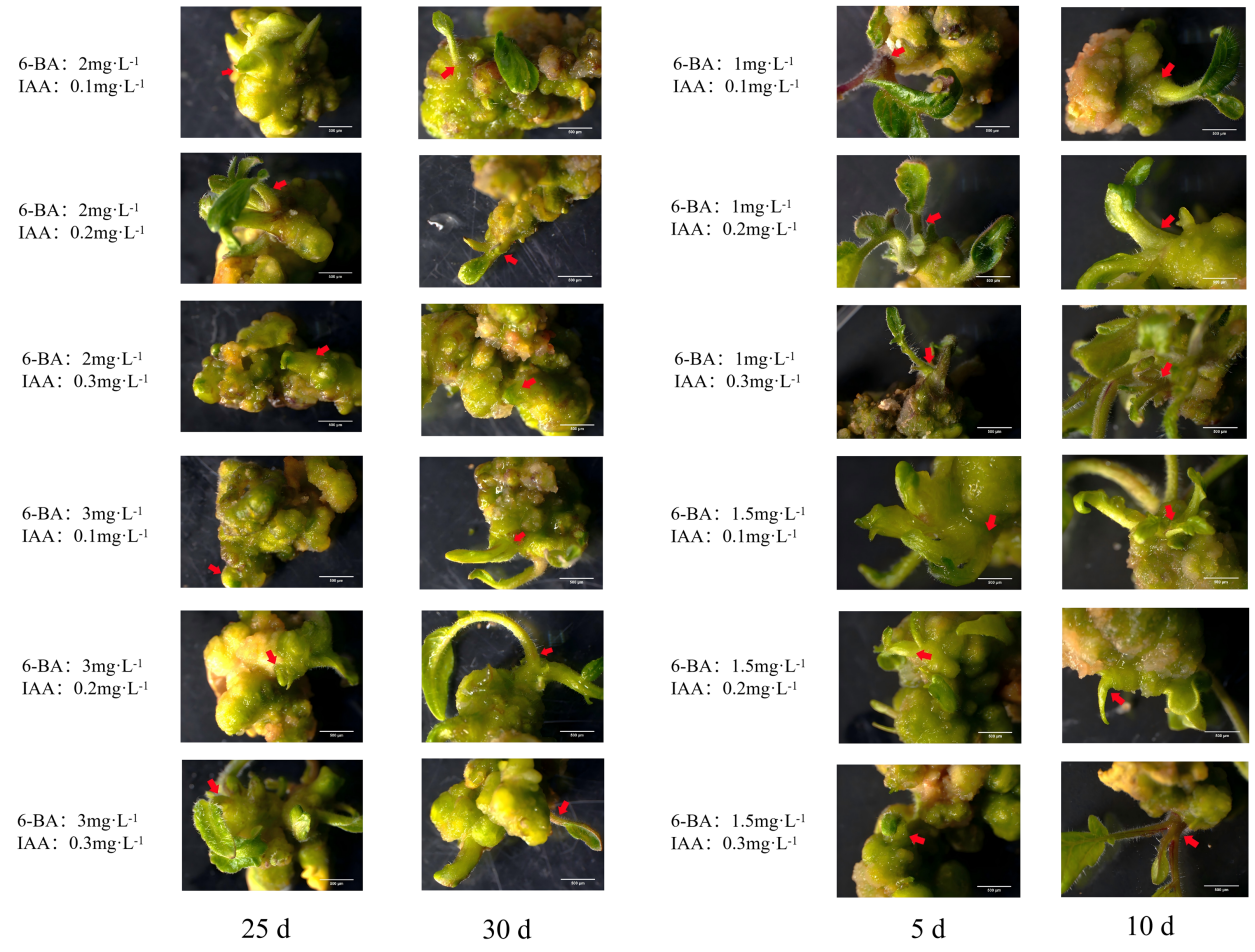
Effects of exogenous 6-BA and IAA concentrations on bud induction and elongation. The explants were inoculated onto MS modified media containing varying concentrations of 6-BA (2 and 3 mg/L) and IAA (0.1, 0.2, and 0.3 mg/L) for 30 days to induce the formation of bud primordia, and were photographed on the 25 th day and the 30th day. The bud is indicated by the red arrow. The explants were then inoculated onto MS modified media containing varying concentrations of 6-BA (1 and 1.5 mg/L) and IAA (0.1, 0.2, and 0.3 mg/L) for 10 days to make the bud primordium elongate, and were photographed on the 5th day and the 10th day. The small shoot is indicated by the red arrow.

The morphological characteristics during the shoot growth stage are shown in Fig. 3. Shoot primordia were visible on all media by day five of the differentiation stage, and both their number and length increased significantly by day 10. By day 10 of the elongation stage, the primordia had developed clearly distinguishable young shoots on all media. Following a comprehensive evaluation, the optimal media for shoot differentiation and elongation were identified as Medium 2 (Table 1; MS media + 2 mg/L 6-BA + 0.2 mg/L IAA) and Medium 8 (Table 2; MS media + 1 mg/L 6-BA + 0.2 mg/L IAA), respectively.

### Effects of exogenous IAA concentrations on *in vitro* rooting and root growth

Elongated shoots were excised and transferred to rooting media containing IAA as the sole exogenous plant growth regulator. The rooting responses across different culture media are summarized in Table 3. By day 21, Medium 14 achieved a rooting rate of 100%, which was significantly higher than those recorded for Medium 13 and Medium 15. By day 28, the rooting rate in Medium 15 increased to 93.33%, with no significant difference relative to Medium 14. Nevertheless, Medium 14 remained superior overall, as it produced the greatest mean root length of 11.76 cm after 28 days, significantly exceeding those observed in Medium 13 and Medium 15. At this stage, the seedlings filled the culture bottles and began to harden. The average hardening rate in Medium 14 was 76.67%, significantly higher than that in Medium 13 and Medium 15.

**Figure 4:**
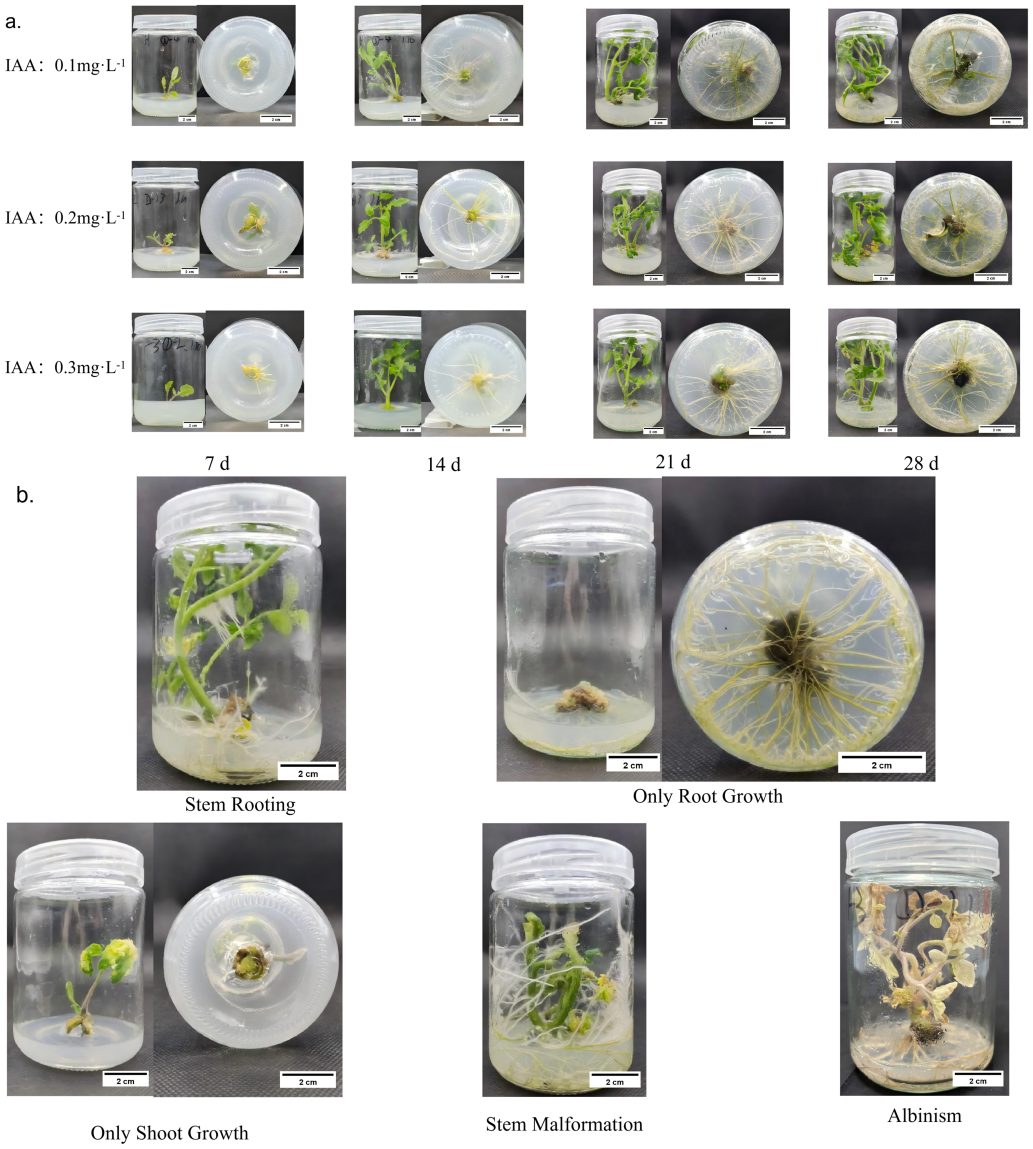
Rooting stages and abnormal growth. The small shoot was cut and inoculated onto MS modified media containing varying concentrations of IAA (0.1, 0.2, and 0.3 mg/L) for 28 days to make the shoot formatted and elongated. The tomato seedlings were photographed every seven days. (A) represents the tomato seedlings that grow normally in each treatment; both the roots and stems grow simultaneously. (B) represents the tomato seedlings that grow abnormally during the rooting stage, with five types.

Morphologically normal plantlets from the three rooting media were selected, acclimatized, and transplanted under standard indoor conditions to assess post-transplant survival. The survival rates from Medium 13, Medium 14, and Medium 15 were 94.44%, 100%, and 88.89%, respectively. The effects of varying IAA concentrations during the rooting stage are illustrated in Fig. 4. In all three media, both the number and length of roots increased gradually with prolonged culture time under standard growth conditions. However, some plants displayed abnormal growth phenomena throughout the rooting culture process. These included stem rooting, root-only growth, stem-only growth, stem malformation, and seedling albinism. Stem rooting and root-only growth were observed across all three media. In contrast, stem-only growth was noted in both Medium 13 and Medium 15. Additionally, stem malformation was exclusively observed in Medium 13, and seedling albinism was exclusively observed in Medium 15. After a comprehensive evaluation of all indicators, Medium 14 was determined to be optimal for the root growth stage, comprising MS media + 0.2 mg/L IAA.

**Table 3:** Rooting rate, root length, and hardening rate.

Medium number	IAA (mg/L)	Rooting rate (%)				Root length (cm)				Hardening rate (%)
		Day 7	Day 14	Day 21	Day 28	Day 7	Day 14	Day 21	Day 28	Day 28
13	0.1	31.11 ± 3.84 ab	66.67 ± 0.00 c	75.55 ± 3.85 c	82.22 ± 3.85 b	1.45 ± 0.11 a	4.88 ± 0.11 c	8.21 ± 0.12 b	9.68 ± 0.29 b	50.00 ± 4.71 b
14	0.2	24.45 ± 3.85 b	95.55 ± 3.85 a	100.00 ± 0.00 a	100.00 ± 0.00 a	0.56 ± 0.03 c	7.06 ± 0.07 a	9.57 ± 0.28 a	11.76 ± 0.38 a	76.67 ± 4.71 a
15	0.3	37.04 ± 3.40 a	88.89 ± 3.84 b	91.11 ± 3.85 b	93.33 ± 6.67 a	0.75 ± 0.09 b	6.13 ± 0.06 b	8.33 ± 0.20 b	9.32 ± 0.25 b	57.78 ± 3.85 b

**Notes.**

Data represent mean ± SD.

Different lowercase letters within a column indicate significant differences (*p* < 0.05).

## Discussion

Tissue culture technology addresses the limitations of traditional breeding methods by improving plant varieties and eliminating external pathogens and genetic variation factors. This approach significantly enhances plant disease resistance and production efficiency. Additionally, tissue culture provides distinct advantages in conserving endangered species, investigating plant developmental mechanisms, and developing novel medicinal resources (Nie et al., 2024).

In tomato, the efficiency of the tissue culture process is influenced by several factors, including genotype, explant source, explant age, medium composition, and environmental conditions (Xu et al., 2025; Yaroshko et al., 2023). Among these variables, genotype is widely recognized as a major determinant of regeneration competence. The Micro-Tom tomato variety is particularly prominent in tomato functional genomics research due to its short stature, relatively brief life cycle, and suitability for high-density planting (Wayase & Shitole, 2014). Numerous cultivated tomato varieties have been successfully subjected to tissue culture, including the Zhongshu series, Lichun, cherry tomatoes, Rekordsmen, and Moryana (Namitha & Negi, 2013; Varlamova et al., 2021).

Explants such as anthers, hypocotyls, cotyledons, stem segments, true leaves, and fruits of tomato can all induce callus and adventitious buds. Among these, cotyledons and hypocotyls exhibit relatively higher induction rates of callus and budding rates of adventitious buds (Mamidala & Nanna, 2011). Additionally, the specific part and developmental stage of the same type of explant significantly influence callus formation. For instance, the green stage of mature fruits from the M82 tomato line is more conducive to callus production than the color-changing and red-ripe stages. Notably, the ascorbic acid content in the callus is also highest during the green stage (Minutolo et al., 2020).

To optimize tomato *in vitro* culture, various bioactive compounds with diverse origins and functions are utilized as growth regulators. These include phytohormones, animal hormones, vitamins, amino acids, antioxidants, and other biologically active additives (Phillips & Garda, 2019; Sidik et al., 2024). Commonly employed exogenous plant growth regulators (PGRs) for tomato *in vitro* culture comprise Indole-3-acetic acid (IAA), α-Naphthaleneacetic acid (NAA), 2,4-Dichlorophenoxyacetic acid (2,4-D), Zeatin (ZT), and 6-Benzylaminopurine (6-BA), aimed at enhancing callus induction and plant regeneration. Other PGRs, including Kinetin (KIN), 6-(γ, γ-dimethylallylamino) purine (2iP), Thidiazuron (TDZ), and Indole-3-butyric acid (IBA) have also been evaluated in tomato regeneration systems (Velda et al., 2023; Zhang et al., 2010). For example, MS medium supplemented with 2 mg/L of 2,4-D and 0.5 mg/L of KIN resulted in the highest callus induction rates of 86.66% for leaves and 70% for hypocotyls in the Roma, Punjab Ratta, and Castle Rock tomato genotypes (Kumar et al., 2017). Similarly, among several apple tomato varieties, the BARI Hybrid Tomato 4 (BH-T4), using 6-BA and NAA, exhibited superior performance in initial culture establishment, callus induction rate, shoot initiation and proliferation, root formation, and acclimatization (Shoyeb et al., 2020).

By adjusting the concentration and combination of exogenous PGRs in the culture medium, adventitious shoots can be effectively induced from various explants across different tomato cultivars (Chirom et al., 2015). In the cultivar Red Rock, the addition of 1.5 mg/L 6-BA was reported to optimize shoot tip proliferation, resulting in an average of 3.4 shoots per explant (Almemry, Ahmed & Bashi, 2021). In contrast, stem explants of BARI tomato-14 cultured on MS medium supplemented with 2 mg/L 6-BA yielded the highest number of shoots, averaging 3.0 shoots per explant after 45 days (Papry, Ahsan & Shahriyar, 2015). In addition to classical PGRs, the incorporation of antibiotics or antioxidants into the culture medium can further enhance the growth of callus and buds in tomato plants. For instance, β-lactam antibiotics have been shown to promote shoot organogenesis, while the antioxidant phenoxane increases both callus weight and rooting frequency (Varlamova et al., 2021; Denysiuk et al., 2024). Collectively, these findings indicate that regeneration efficiency in tomato is highly dependent on the precise hormonal and nutritional composition of the culture medium, and that medium optimization must be tailored to genotype, explant type, and developmental stage.

In the present study, the culture media were supplemented with different concentrations of 6-BA and IAA to stimulate different developmental stages in tomato explants. During the callus induction stage, the medium containing 0.2 mg/L IAA and 2 mg/L 6-BA exhibited notable advantages, achieving a callus formation rate of 89% at day 15 and of 97% at day 20, along with an embryogenic cell proportion of 63.88%. These results support the view that a moderate concentration of cytokinin (6-BA) combined with a low concentration of auxin (IAA) provides a favorable hormonal environment for callus proliferation and acquisition of embryogenic competence. Embryogenic cells represent undifferentiated cells that possess the potential for embryonic development. These cells are typically found in callus or suspension culture systems. Although complete embryonic structures may not yet be morphologically apparent, the abundance of embryogenic cells within callus tissue is a key indicator of regenerative potential (Fan et al., 2024). Previous studies have similarly shown that regeneration efficiency in tomato can be enhanced by alternative developmental routes; for example, culture at pH of 4.0, along with high concentrations 6-BA and thidiazuron, has been shown to promote the formation of new rhizoid tuber structures in tomato callus, thereby enhancing plant regeneration efficiency through somatic embryogenesis (Saeed et al., 2019). Likewise, other methods have been identified to increase the rate of somatic embryogenesis. For instance, employing CRISPR-activation systems to edit the WRKY29 gene in cotyledon explants of Micro-Tom tomato significantly improved their somatic embryogenesis efficiency (Valencia-Lozano et al., 2024).

In the present study, the medium containing 3 mg/L 6-BA significantly enhanced callus formation rates during the early stage (day 10). However, a notable decline was observed in the mid to late stages. This decline may be attributed to the impact of elevated cytokinin concentrations on critical browning enzymes, including polyphenol oxidase, peroxidase, and phenylalanine ammonia-lyase (Permadi et al., 2024). Histological analysis further showed that the medium supplemented with 0.1 mg/L IAA and 3 mg/L 6-BA produced the highest embryogenic cell rate (67.66%). However, the subsequent reduction in callus formation rate suggests that while short-term exposure to high cytokinin concentrations may promote embryogenic cell differentiation, it is essential to adjust the hormone ratio promptly to sustain tissue viability.

During the shoot induction stage, the medium containing 0.2 mg/L IAA and 2 mg/L 6-BA demonstrated a significant advantage, achieving a 63% budding rate by day 30 (Table 1). This positive correlation, coupled with its strong performance during the callus stage, underscores the importance of high-quality callus as the foundational material for subsequent organ differentiation. Notably, the medium with 0.3 mg/L IAA and 3 mg/L 6-BA produced the highest budding rate of 41.33% at day 25. However, this was later surpassed by the medium containing 0.2 mg/L IAA and 2 mg/L 6-BA. This phenomenon suggests that the high concentration of cytokinin accelerates the initiation of shoot primordia but inhibits their subsequent development (Zhang & Finer, 2016). During the shoot elongation stage of this study, the medium with 0.2 mg/L IAA and 1 mg/L 6-BA exhibited optimal efficiency. This finding suggests that a reduction in cytokinin concentration favors shoot development, consistent with the classical “hormone ratio theory,” which posits that a low cytokinin/auxin ratio promotes shoot elongation (Melnyk, 2023).

During the rooting stage, the medium supplemented with 0.2 mg/L exogenous IAA demonstrated a distinct “late-advantage” effect on the development of adventitious roots (Table 3). This delayed pattern may be attributed to the dose-dependent response of tomato adventitious roots to exogenous IAA (Da Costa et al., 2013). In comparison to high-concentration IAA treatments, 0.2 mg/L IAA provided a more consistent effect on root development. Although this treatment group exhibited the lowest rooting rate on day 7, its rooting performance improved rapidly after day 14, ultimately achieving a 100% rooting rate and an average root length of 11.76 cm. Specifically, the high-concentration IAA group displayed a significant rooting advantage in the early stages; however, this promoting effect diminished considerably with extended culture duration. Moreover, the 0.2 mg/L IAA formulation achieved the highest hardening rate at 76.67%, which correlated directly with its lower deformity rate. This finding suggests that an appropriate concentration of IAA can facilitate the balanced development of both aerial and underground parts, while excessively high concentrations may disrupt physiological processes and lead to abnormalities, such as the formation of shoot-born roots.

Despite the establishment of an efficient and stable tissue culture system in this study, several limitations should be addressed in future research. First, this study concentrated on optimizing the concentration ratio of exogenous PGRs, specifically 6-BA and IAA, for each *in vitro* culture stage. However, the regulatory roles of endogenous auxin were not fully investigated. Subsequent research will focus on clarifying the regulatory mechanism that governs the interplay between endogenous and exogenous auxin *in vitro* regeneration of processing tomato. Additionally, the regeneration protocol established here provides a strong foundation for the development of an Agrobacterium-mediated genetic transformation system, which will facilitate subsequent molecular breeding of innovative processing tomato cultivars.

## Conclusions

The stage-specific adjustment of exogenous PGRs significantly enhanced the regeneration efficiency of the processing tomato cultivar ‘Ligeer 87-5.’ Based on the results obtained, a staged medium approach is recommended throughout the entire tissue culture process. For callus induction, the medium supplemented with 0.2 mg/L IAA and 2 mg/L 6-BA was optimal. The same formulation was also most effective during the early stage of shoot differentiation for rapid primordium initiation. After 10 days, transferring the regenerating tissues to MS medium containing 0.2 mg/L IAA and 1 mg/L 6-BA promoted shoot development and elongation. For root induction, the MS medium supplemented with 0.2 mg/L IAA facilitated plantlet establishment. Overall, this study established an efficient and reproducible *in vitro* regeneration system using true leaf explants of the processing tomato cultivar ‘Ligeer 87-5.’ The findings provide a valuable reference for optimizing regeneration systems for other processing tomato cultivars. The results of this study also offer reliable technical support for the genetic improvement of processing tomatoes.

##  Supplemental Information

10.7717/peerj.21520/supp-1 Supplemental Information 1 Callus Induction Rate of Tomatoes at Different Times The main statistics recorded were the number of explants with induced callus in tomato at various time points.

10.7717/peerj.21520/supp-2 Supplemental Information 2 The budding rate of tomatoes at different times The main statistics recorded were the number of callus pieces with developed buds in tomato at various time points.

10.7717/peerj.21520/supp-3 Supplemental Information 3 Embryogenic Rate of Tomato Callus on the 20th Day The main statistics recorded were the number of embryogenic cells and non-embryogenic cells observed in tomato callus under paraffin sections on day 20.

10.7717/peerj.21520/supp-4 Supplemental Information 4 The number of plants that reached the hardening and transplantation stage The Hardening Rate , which is the percentage of tomato plants that met the hardening and transplantation requirements after acclimatization out of the total number of plants.

10.7717/peerj.21520/supp-5 Supplemental Information 5 Root Length of Tomato Callus under Different Conditions and Different Formulations at Different Times The main statistics recorded were the root length induced from tomato callus under different time points and medium formulations.

10.7717/peerj.21520/supp-6 Supplemental Information 6 Rooting Rate of Tomato Callus under Different Conditions and Different Formulations at Different Times The main statistics recorded were the number of rooted plants induced from tomato callus under various time points and medium formulations .

## Additional Information and Declarations

### Data Availability

The following information was supplied regarding data availability: The raw data are available in the Supplemental Files.
